# The Peritubercle Lucency Sign as a Considerable Predictive Factor for Contralateral Hip Slippage in Unilateral Slipped Capital Femoral Epiphysis Cases

**DOI:** 10.1055/s-0044-1790578

**Published:** 2024-12-21

**Authors:** Anastácio Kotzias Neto, Renan Vinicius Romano Martinelli, Marthina Alice Gressler, Marco Aurélio de Oliveira

**Affiliations:** 1 Departamento de Ortopedia Pediátrica, Hospital Infantil Joana de Gusmão, Florianópolis, SC, Brasil

**Keywords:** early diagnosis, hip, radiography, slipped capital femoral epiphysis

## Abstract

**Objective** To determine whether the radiographic parameter at the epiphyseal tubercle region (peritubercle lucency sign) on the unaffected side can predict slipped capital femoral epiphysis (SCFE).

**Methods** We retrospectively reviewed patients who received an initial diagnosis of unilateral SCFE between 1995 and 2020 at a pediatric hospital in a Brazilian state's capital. The patients were monitored for at least 18 months. Two reviewers independently and blindly assessed the radiographs for the presence or absence of the sign. Disagreements were resolved by a third senior reviewer.

**Results** Out of the 115 cases reviewed, the peritubercle lucency sign was observed in 21 of the 30 patients who developed the disease in the contralateral hip. The sign was observed on an average of 21 days after the diagnosis on the initial side, and approximately 301 days prior to the condition affecting the contralateral hip. It was present in 95% and 85% of the cases on the lateral (frog-leg) and anteroposterior (AP) views, respectively. Interobserver reliability was measured using the Kappa test (k = 0.0801). There was a significant relationship between the presence of the sign and SCFE ( *p* < 0.001).

**Conclusions:** We propose that the peritubercle lucency sign can be used as a supplementary tool in early diagnosis, for it is beneficial in the therapeutic planning.

**Level Of Evidence:** Level III – Diagnostic Study In Nonconsecutive Patients (Without Consistently Applied ‘Gold Standard’ As Reference)

## Introduction

During the rapid growth phase of adolescence, increased fragility and shear stress can result in the slippage of the capital femoral epiphysis off the femoral neck, a condition known as slipped capital femoral epiphysis (SCFE). 1 2 The exact pathophysiology remains unclear, but the epiphyseal tubercle is thought to play a crucial role in disease development. The suggested mechanism involves a rotation during SCFE, so the tubercle would act as a fulcrum located eccentrically in the posterosuperior quadrant of the physis. 3 This is the most pronounced bony structure observed on the physeal surface of the capital femoral epiphysis. 4 Liu et al. 5 suggested that the epiphyseal tubercle is primarily responsible for stabilizing the capital femoral epiphysis and safeguarding the lateral epiphyseal vessels. During adolescence, the tubercle undergoes a reduction in height and perimeter, potentially leading to local instability and an increased risk of necrosis. 6 The disease has an incidence rate of 1 to 7 cases per 100 thousand people, and it predominantly affects boys, typically around the age of 14. 7 Skeletal maturity, metabolic disorders, femoral morphology, and body mass index can influence the development of the disease, which is often associated with increased body weight. 8 9 The disease more frequently impacts the left side and may affect both sides in up to 80% of the cases. It can occur simultaneously or at different times, usually within the first 18 months after the occurrence on one side. 10 11 Surgical intervention is a well-established treatment for the disease, and monitoring of the contralateral hip is crucial. In recent years, various radiographic parameters have been examined to identify early signs of SCFE in the contralateral hip; they include the Southwick angle, which indicates increased epiphyseal inclination, 12 the posterior inclination angle, 13 the alpha angle, 14 and the epiphyseal inclination. 15 While some authors 16 17 advocate for prophylactic fixation based on a combination of clinical data, radiographic evidence, and social indicators, assessment of the unaffected hip remains a subject of study. 18 Recently, a new objective imaging parameter, known as the peritubercle lucency sign, has been proposed. 19 This sign is believed to be evident on radiographs since the first changes that occur in the epiphyseal tubercle and the corresponding metaphysis. However, its practical application in clinical settings remains uncertain.

The present study aims to determine whether the peritubercle lucency sign could be used as a reliable radiographic parameter for early diagnosis and as a predictor of disease in the contralateral hip among patients with unilateral SCFE. Additionally, we aim to assess if the absence of this sign can serve as a predictor of the absence of SCFE. Finally, the study evaluates the interobserver agreement in radiographic analyses.

## Materials and Methods

**Fig. 1:**
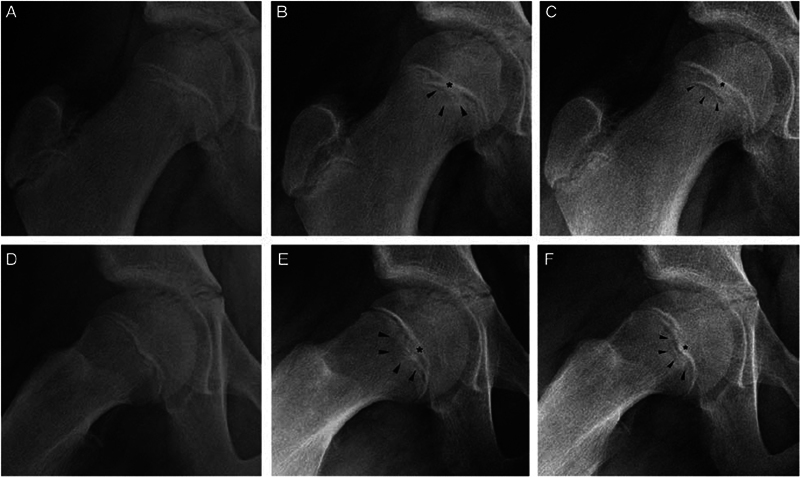
Peritubercle lucency sign 19 .

The current retrospective longitudinal study was conducted at Hospital Infantil Joana de Gusmão, in the city of Florianópolis, State of Santa Catarina, Brazil. The study population was composed of patients initially diagnosed with unilateral SCFE who had not previously undergone surgery on the opposite side and had a minimum outpatient follow-up of 18 months with the Orthopedics Service between 1995 and 2020. Patients who did not exhibit physeal closure during this period were monitored until the complete closure of the triradiate cartilage, ensuring that all were followed up until they attained skeletal maturity. The selected patients were numbered sequentially according to their inclusion in the study. Data were collected retrospectively from electronic and physical medical records, according to the research instrument described in Annex 1 . Radiographs obtained during the follow-up period were classified chronologically for each case and reviewed by two early-career orthopedic surgeons (reviewers 1 and 2). They searched for the peritubercle lucency sign as described in 2018 by Maranho et al. 19 ( Fig. 1 ). Only radiographs taken in the anteroposterior (AP) and lateral (frog -leg) views were considered valid. Reviewers 1 and 2 assessed the presence or absence of the sign in all radiographs. The sign was considered present if it appeared in at least one radiograph, and absent if it was not found in any. The responses were considered valid when both reviewers agreed on the presence or absence of the sign. In cases of disagreement, a third evaluator, a senior orthopedic surgeon referred to as reviewer 3, was consulted for a final analysis. All reviewers conducted their analyses independently and blinded. Previously, all 3 reviewers completed intra- and interobserver reliability tests, conducted in 2 rounds, analyzing 10% (15/115 cases) of the total sample size.

**Annex 1:** Research instrument used in the study

Name
Medical record
Date of birth – day/month/year
Age – in years
Sex – male/female
City of origin
Comorbidity – description
Percentile on the weight x age chart
Time of symptom evolution – in days
Trauma – present or absent
Location of the onset of symptoms – description
Date of first evaluation – day/month/year
First side – right/left
Degree of the first side – 0/1/2/3
Surgery in the first side – day/month/year
First side: fixation type – *in situ* /reduction
First side: material – description
Second side – right/left
Degree of the second side – 0/1/2/3
Second side: fixation type – *in situ* /reduction
Second side: material – description
Lucency sign – present/absent
Sign presence – yes/no
Follow-up time – in months
Complication – description
Material withdrawal – yes/no

**Fig. 2:**
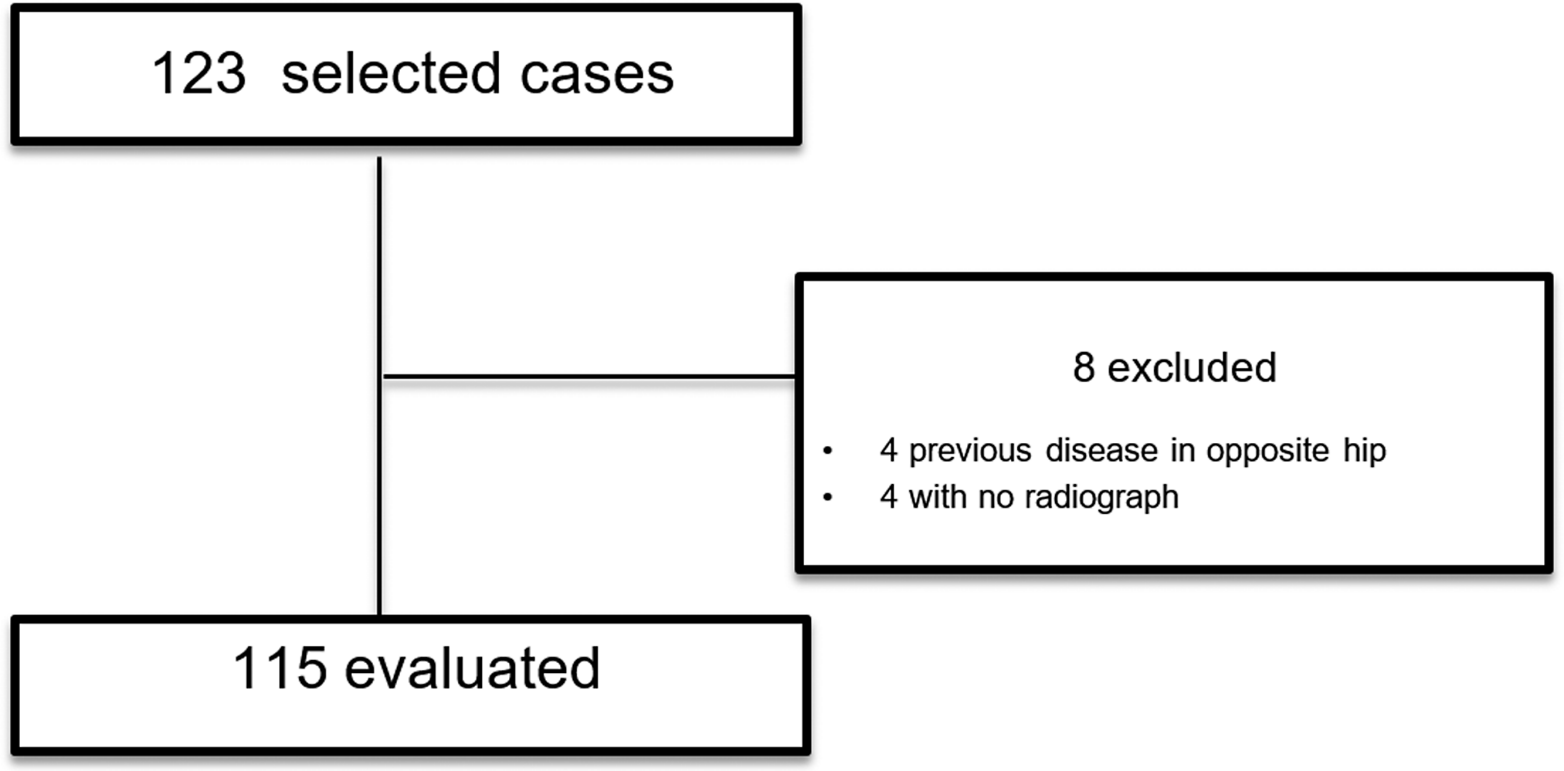
Flowchart of the patient selection process. A total of 115 cases were selected based on the inclusion criteria.

We excluded patients who had previously undergone fixation in a different service or prophylactic fixation of the unaffected hip, as well as those who did not have adequate radiographs for review and those who were diagnosed with another disease in the contralateral hip. Cases that have already presented disease in both hips from the beginning (bilateral) were not considered. A flowchart of the patient selection process is shown in Fig. 2 .

The variables were submitted to descriptive analyses. The relationships among the variables of interest were examined using the contingency coefficient C correlation test, with a significance level of *p* < 0.01. The interobserver agreement was verified using the Kappa coefficient of agreement, with a significance level of *p* < 0.01. Data were analyzed using the IBM SPSS Statistics Subscription for Windows, version Build 1.0.0.1406 (IBM Corp., Armonk, NY, United States).

The current study was conducted after we received approval from the Institutional Research Ethics Committee (opinion no. 42937121.2.0000.5361). The study was based on Resolution no. 500/16 of the Brazilian National Health Council (Conselho Nacional de Saúde, CNS, in Portuguese), and it adhered to the ethical principles of beneficence, non-maleficence, justice, and autonomy.

## Results

The present study included 115 patients diagnosed with unilateral SCFE at their first consultation. During the follow-up, 30 patients developed the condition in the contralateral hip. The average age was of 11.81 (range: 9–15) years, and the average follow-up period was of 32.8 (range: 4–96) months ( Table 1 ). For the obesity and overweight analysis, we used the weight-for-age index as a benchmark, referencing the percentile charts from the Centers for Disease Control and Prevention. 20 Two patients had multiple comorbidities (hypothyroidism and conditions associated with obesity). Regarding the duration of symptoms, we found that 33% of cases were acute, 62% were chronic, and 5% were chronic-acute, as per the Fahey and O'Brien classification. 21 The severity of each affected hip was measured according to the quantification classification of the slippage of the epiphysis relative to the femoral neck, as described by Wilson et al. 22 Of the two cases with a severity rating, one was diagnosed through scintigraphy, and the other was diagnosed based on pain symptoms and refusal to bear weight on the affected limb. The average time from the first consultation to the initial surgical procedure was 3.19 of (range: 0–30) days. For patients who, during follow-up, developed the disease in the contralateral hip and required fixation, the interval between the two surgeries was on average 312 (range: 26–810) days. In-situ fixation was the method of choice in 86.89% of the cases. The materials used for fixation were either a cannulated screw or a threaded metallic wire (Schanz pin). The choice of implant varied over the years, based on when the procedure was performed and the availability of the synthesis materials at the hospital. The evaluation of the peritubercle lucency sign is outlined in Table 2 and the sign was found to be present in 31.3% of all cases. Out of the 30 individuals who developed the disease, 21 exhibited this sign. The youngest patient who presented the sign and progressed to the disease was a male aged 9 years and 1 month, and the oldest patient was a boy aged exactly 14 years. When pairing the presence or absence of the sign with age, the null hypothesis must be retained since age behavior does not differ significantly between the groups that did or did not progress to the disease ( Table 3 ). A significant correlation between the presence of the peritubercle lucency sign and contralateral SCFE was found ( *p* < 0.001). The contingency coefficient C showed a *p* -value of 1.06 × 10 ^−7^ ( *p* < 0.001). The upper limit of the contingency coefficient C is 0.707, and the correlation is 0.44. The data obtained from these analyses are described in Table 4 .

**Table 1:** Demographics and clinical characteristics of the study sample

	n	Valid percentage**
Sex		
- Male	76	66.1%
- Female	39	33.9%
**Comorbidity**		
- Obesity	66	56.4%
- Overweight	17	20%
- Others	3	3.6%
- Degree*		
0	2	
1	68	
2	26	
3	15	
- Type of fixation		
Reduction	14	
In-situ fixation	99	
**Second affected hip**		
- Side		
Right	23	76.7%
Left	7	23.3%
- Degree		
0	11	
1	15	
2	1	
3	0	
- Type of fixation		
Reduction	0	
In-situ fixation	28	

**Note:** *According to Wilson et al. 22

**Note:** **Valid percentage only considers cases that contained information in the medical record.

**Table 2:** Evaluation of the peritubercle lucency sign

Lucency Sign	Unilateral (n)	Bilateral (n)	Total (N)
Absent	71	9	80
Present	14	21	35
**Total**	**85**	**30**	**115**

**Notes:** Unilateral – there was no evolution to disease; bilateral – there was evolution to disease.

3.1 Patients with the sign: pairing the ages of those affected (bilateral( and not affected (unilateral)

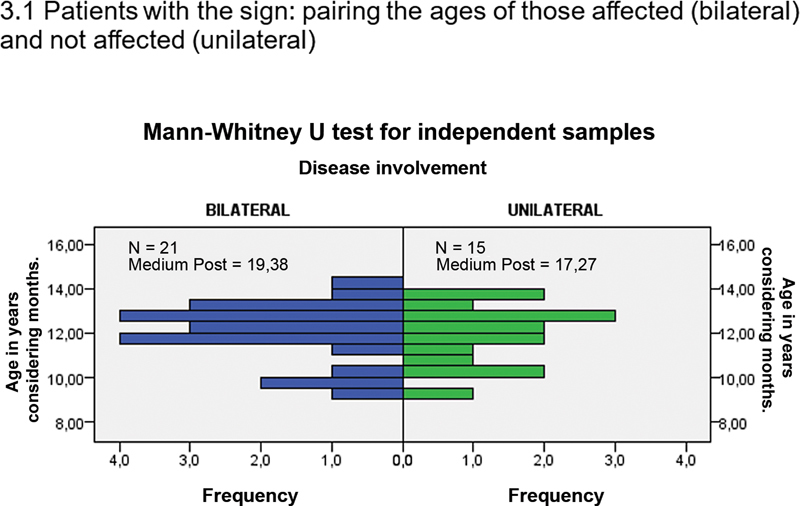



**Table 3:** Pairing sign with age

	N	Minimum	Maximum	Average	Deviaton error
Age (months)	36	9.08	14.00	11.9844	1.33053

3.2 Patients without the sign: pairing the ages of those affected (bilateral) and not affected (unilateral)

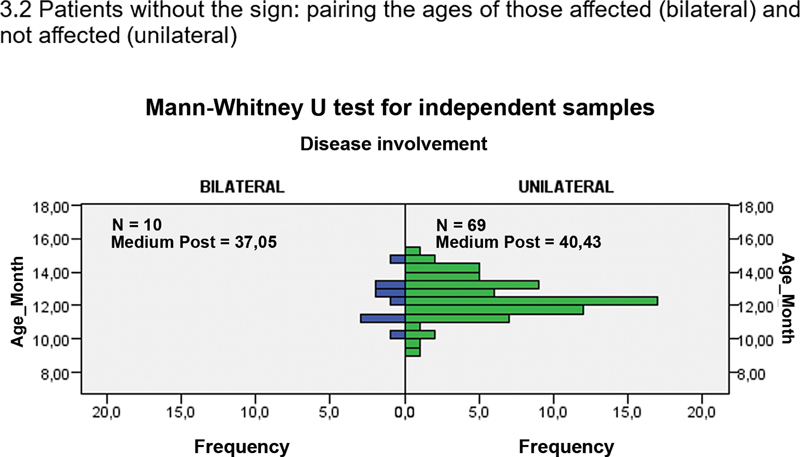



**Table:** 

	N	Minimum	Maximum	Average	Deviaton error
Age (months)	79	9.00	15.42	12.4181	1.20627

3.3 Group with all unaffected patients (unilateral): pairing the ages of those with and without the sign

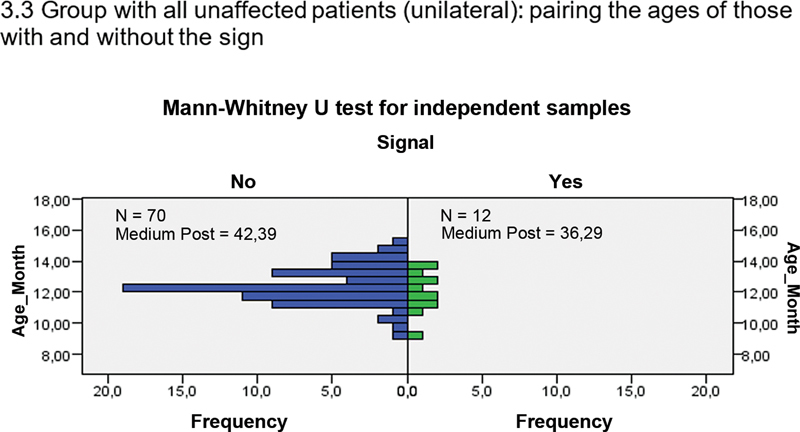



**Table:** 

	N	Minimum	Maximum	Average	Deviaton error
Age (months)(	82	9.00	15.42	12.3309	1.22065

3.4 Group with all affected patients (bilateral): pairing the ages of those with and without the sign

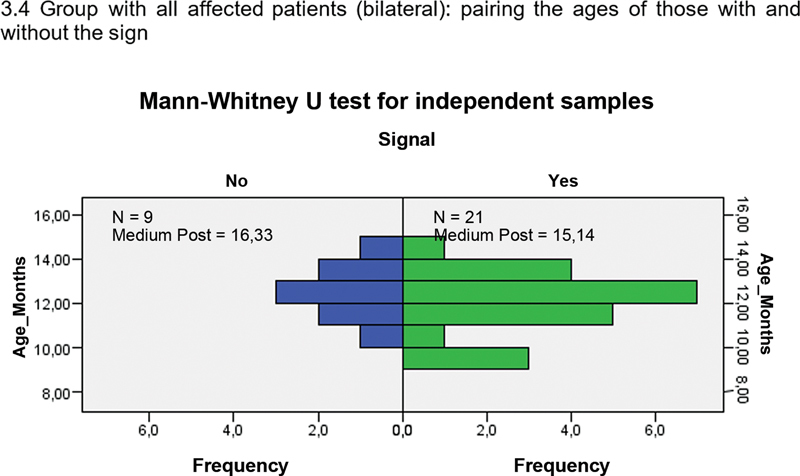



**Table:** 

	N	Minimum	Maximum	Average	Deviaton error
Age (months)	30	9.08	14.67	12.1640	1.35121

**Table 4:** Statistical analysis: association between the presence of the peritubercle lucency sign and development of the SCFE

Statistical data	
Sample (N)	115
Sign emergence time	1 month
Time before the slippage	40 weeks
Anteroposterior/Frog-leg radiographs	85%/95%
Accuracy	80%
Sensitivity	70%
Specificity	83%
Positive predictive value	60%
Negative predictive value	89%

**Fig. 3:**
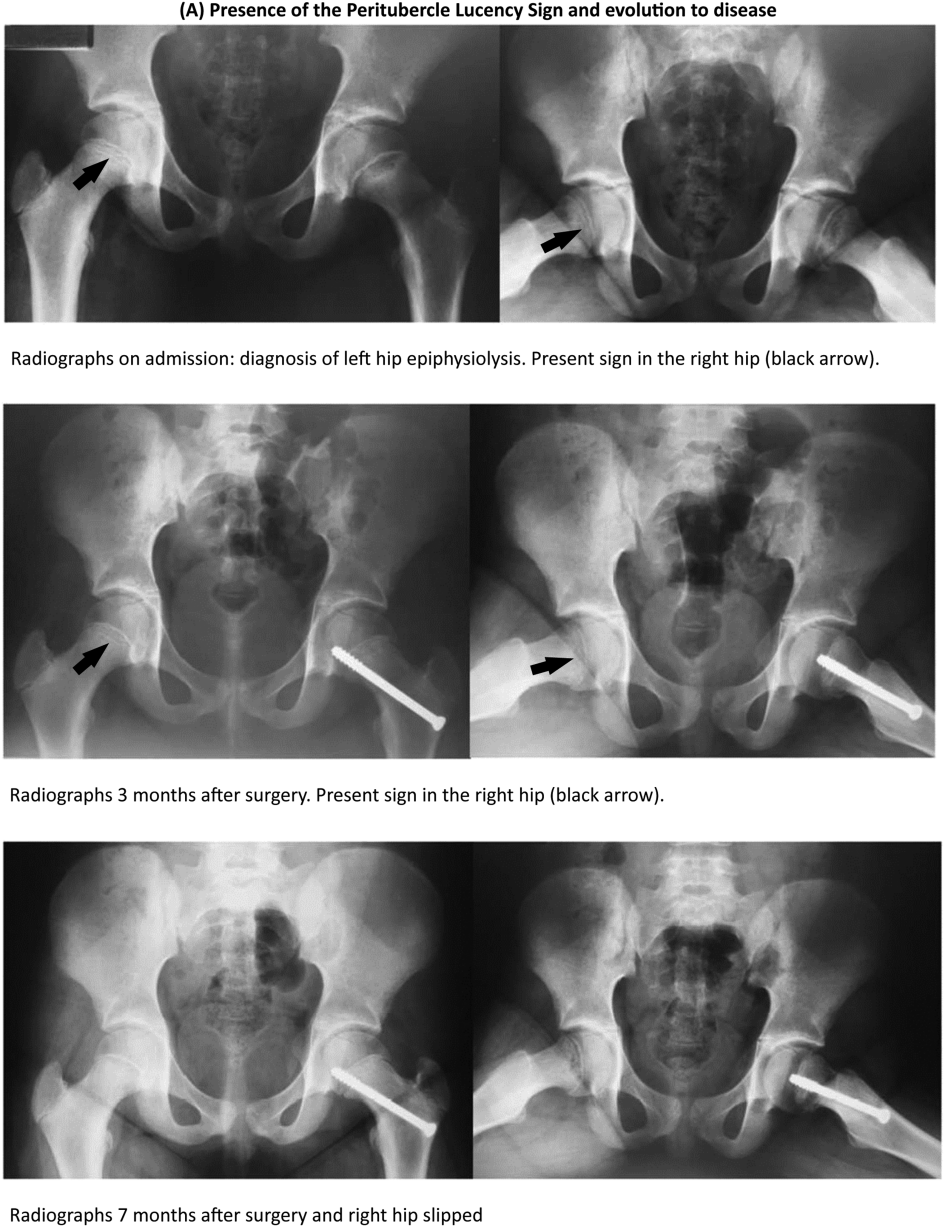
Radiological assessment of peritubercle lucency sign and development of slipped capital femoral epiphysis (SCFE). ( **A** ) Presence of the peritubercle lucency sign and development of the disease; ( **B** ) presence of the peritubercle lucency sign and absence of the disease; ( **C** ) absence of the peritubercle lucency sign and development of the disease; and ( **D** ) absence of the peritubercle lucency sign and absence of the disease.

In cases in which the sign was present and the patients developed the disease in the contralateral hip, the sign was observed on average 21 days after the disease was first diagnosed on the initial side. The slippage typically occurred approximately 301 days later. In this group, the sign was more frequently observed in the frog-leg view (20/21 cases, 95%) than in the AP view (18/21 cases, 85%). The inter-observer agreement between reviewers 1 and 2 was found to be strong, as measured by the Kappa test (k = 0.0801). In 10 cases, the evaluations needed to be reviewed by reviewer 3. No significant correlation was identified in the analysis regarding the presence or absence of the sign and the variables of interest ( Table 5 ). It is important to note that, despite the visual differences observed between the groups based in terms of the presence or absence of the sign, these differences did not show statistical significance. Eight cases were excluded for the following reasons: two had previously undergone fixation at a different facility; two had a degenerative disease in the contralateral hip (Legg-Calvé-Perthes disease); and four were excluded since follow-up control radiographs were not available. We were able to identify the presence and absence of the sign, with or without development of the disease, as shown in Fig. 3 .

**Table 5:** Statistical analysis regarding the presence or absence of the sign and the variables of interest

Variables of interest compared	Statistical test	Degree	Significance	Conclusion
Present sign versus age	1.073	1	0.376	Does not reject H _0_
Present sign versus sex	3.196	1	0.091	Does not reject H _0_
Present sign versus comorbidity	4.487	2	0.106	Does not reject H _0_
Present sign versus laterality	1.073	1	0.376	Does not reject H _0_
Present sign versus degree of the first side	3.785	2	0.151	Does not reject H _0_

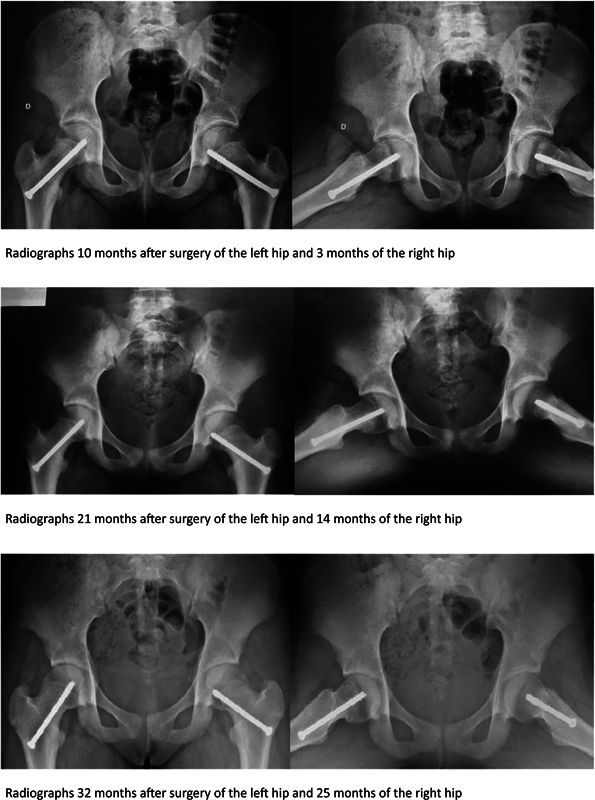

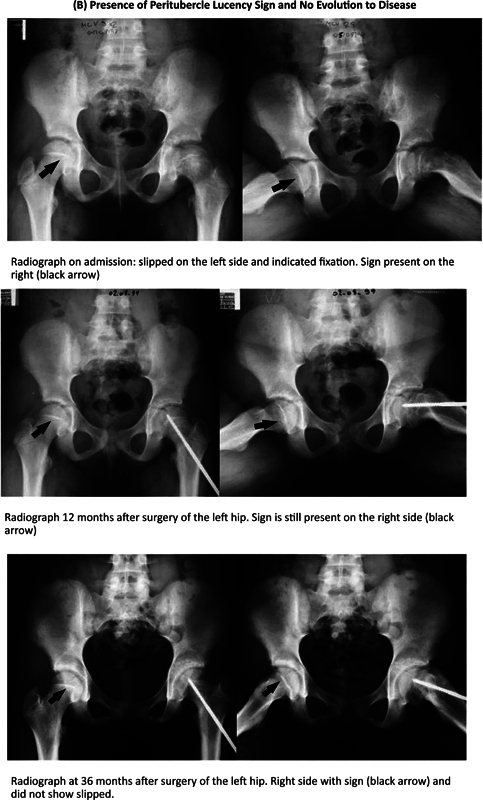

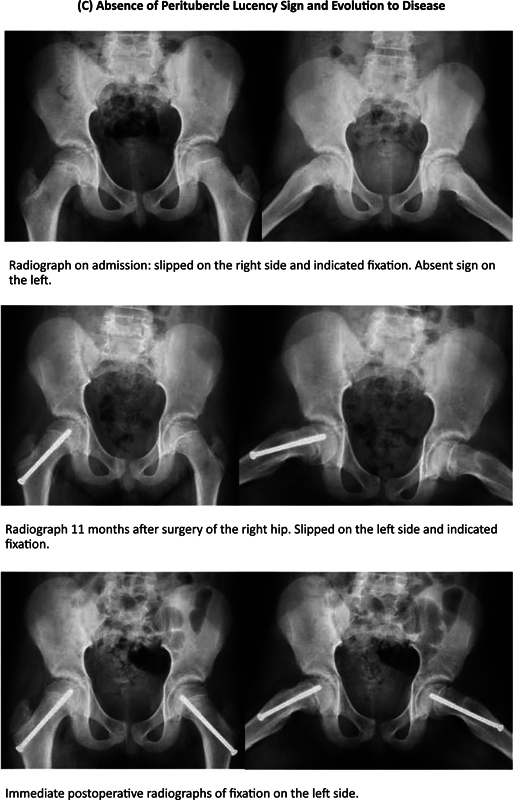

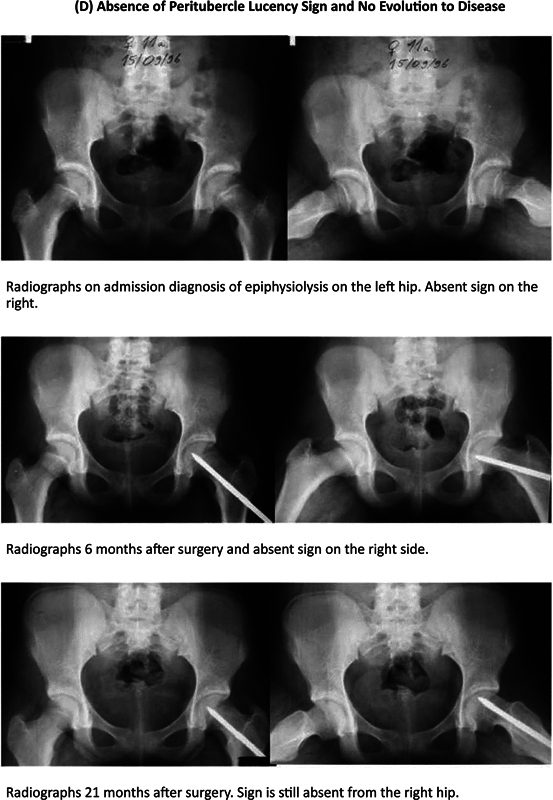

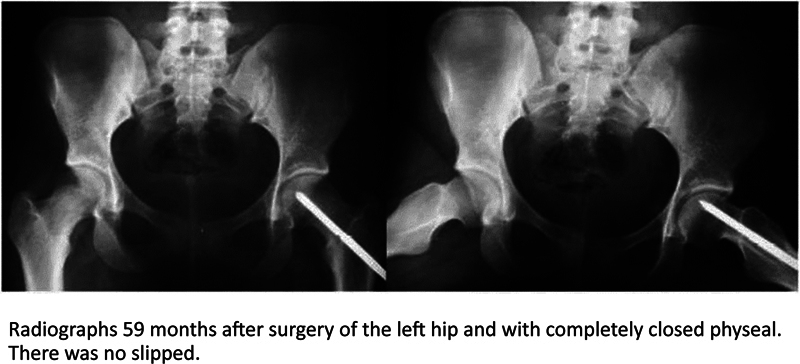











## Discussion

While anatomical and histological alterations in the disease have been extensively described in previous studies, 2 3 the peritubercle lucency sign on radiographs was only recently proposed as an indicator for early diagnosis. 19 This method has yielded results that are comparatively superior to those of magnetic resonance imaging. 23 The tubercle is commonly located in the posterosuperior quadrant, positioned more posteriorly in younger children and superiorly in older ones. 5 Its primary role is to provide structural support against the shear forces acting on the capital femoral physis. 5 The radiographic manifestation of the action of these forces would be the peritubercle lucency. 19

As described by Kleinman et al., 6 abnormalities in the capital femoral metaphysis (juxtaphyseal) can be challenging to detect, likely reflecting the localized reparative response to stress mechanisms that weaken the area. Song 24 suggested that orthopedists should explore new methodologies and algorithms to facilitate earlier diagnosis and treatment of this condition.

The present study aimed to assess the applicability of this sign. In our assessment, of 115 patients, all of whom met the inclusion criteria, 30 developed SCFE on the contralateral hip. The sign was present in 31% of the total sample (36/115). Of these 36 patients, 58% (21/36) developed the disease, while 42% (15/36) remained with the unilateral affection. In cases in which the sign was considered absent (79/115), the majority (70/79) did not develop SCFE, and 11% (9/79) developed the disease. Upon analyzing the 30 patients that developed SCFE, we found the sign present in 70% of these cases (21/30). In contrast, among the 85 cases that remained exclusively unilateral (that is, they did not develop SCFE) the absence of the sign was noted in 82% of these patients (70/85).

Our sensitivity and specificity indices were of 70% and 82% respectively. While these values are relatively lower than those reported by Maranho et al., 19 they are still considerably high. In 80% of the cases, we observed the sign on the initial radiographs of the first affected side either in the pre- or immediate postoperative period. The Kappa index between the two main observers reached a level of strong and superior agreement, according to Cohen. 25 However, to enhance the reliability of the findings, a third senior observer conducted additional analyses.

Our analysis had some limitations regarding the accessibility of previous radiographs and the lack of information in certain medical records. Despite these constraints, we maintained a minimum follow-up period of 18 months, as this time frame is widely accepted in the literature for the expected occurrence of SCFE in the contralateral hip. 1 9 10 Although there are documented cases in which SCFE occurred after this period, we did not encounter any cases in this study. Such cases were monitored until the complete closure of the triradiate cartilage was achieved.

Another limitation was the absence of a standardized position of patients during radiography. The position, which could vary in degrees of flexion, extension, rotation, and abduction, often depended on the patient's level of pain. However, the frog-leg view, which we considered the most effective for diagnosing SCFE, was less compromised and was the one that most often revealed the presence of the sign.

We believe that seeking and verifying the peritubercle lucency sign serves as a useful guide for early diagnosis, 26 helping to prevent more severe and pronounced cases. Further studies need to be conducted to determine whether the forces acting on the capital femoral physis can be considered responsible for the sign.

## Conclusion

Our findings suggest a significant correlation between the presence of the sign on radiographs and the development of the disease in the patient's contralateral hip. While there are more accurate diagnostic tests, such as magnetic resonance imaging, the presence of the sign on radiographs appears to predict disease development. The interobserver agreement was similar to that of other studies, supporting its applicability in the clinical practice. Hence, the peritubercle lucency sign emerges as a promising supplementary tool in the early diagnosis of SCFE, being useful for therapeutic planning and feasible for wide-scale application when high-cost complementary exams are not available. While we found no connection involving the sign and patient-specific characteristics or disease traits, we advise using it judiciously, considering the clinical examination and, if required, other complementary imaging tests. This is because SCFE may still occur, even in the absence of the sign.
