# Supplementary material for: The Peritubercle Lucency Sign as a Considerable Predictive Factor for Contralateral Hip Slippage in Unilateral Slipped Capital Femoral Epiphysis Cases
Source: Rev Bras Ortop (Sao Paulo). 2024 Dec 21;59(6):e922–35. doi: 10.1055/s-0044-1790578 (PMC11663065; doi:10.1055/s-0044-1790578)
Supplement: Related texts

# Research article: O sinal de lucência peritubercular como fator preditivo considerável de escorregamento no quadril contralateral em casos de escorregamento epifisário unilateral da cabeça do fêmur

**Keywords:** diagnóstico precoce, escorregamento das epífises proximais do fêmur, quadril, radiografia

## Abstract

**Objetivo** Determinar se o parâmetro radiográfico na região do tubérculo epifisário (sinal de lucência peritubercular) no lado não acometido pode prever o escorregamento epifisário da cabeça do fêmur (EECF).

**Métodos** Revisamos retrospectivamente pacientes com diagnóstico inicial de EECF unilateral entre 1995 e 2020 em um hospital pediátrico de uma capital brasileira. Os pacientes foram monitorados por pelo menos 18 meses. Dois revisores avaliaram as radiografias de forma independente e cega quanto à presença ou ausência do sinal. As divergências foram resolvidas por um terceiro revisor sênior.

**Resultados** Dos 115 casos revisados, o sinal de radiotransparência peritubercular foi observado em 21 dos 30 pacientes que desenvolveram a doença no quadril contralateral. O sinal foi observado em média 21 dias após o diagnóstico no primeiro lado e aproximadamente 301 dias antes do acometimento do quadril contralateral. Esteve presente, em 95% e 85% dos casos nas incidências em perfil (perna de rã) e anteroposterior (AP), respectivamente. A confiabilidade interobservador foi medida pelo teste Kappa (k = 0,0801). Houve relação significativa entre a presença do sinal e o EECF ( *p* < 0,001).

**Conclusão** Propomos que o sinal de lucência peritubercular seja utilizado como ferramenta complementar no diagnóstico precoce de epifisiólise do fêmur proximal, pois é benéfico no planejamento terapêutico.

**Nível De Evidência** Nível III – Estudo De Pacientes Não Consecutivos (Sem Um “Padrão-Ouro” Aplicado Consistentemente Como Referência).

## Introdução

Durante a fase de rápido crescimento da adolescência, o aumento da fragilidade e o estresse de cisalhamento podem provocar o escorregamento da epífise da cabeça do fêmur para fora do colo femoral, uma doença conhecida como escorregamento epifisário da cabeça do fêmur (EECF). 1 2 A fisiopatologia exata ainda é obscura, mas acredita-se que o tubérculo seja crucial no desenvolvimento da doença. O mecanismo sugerido envolve uma rotação durante o EECF e, assim, o tubérculo atuaria como um fulcro de localização excêntrica no quadrante posterossuperior da fise. 3 Esta é a estrutura óssea mais pronunciada observada na superfície fisária da epífise da cabeça do fêmur. 4 Liu et al. 5 sugeriram que o tubérculo epifisário é o principal responsável pela estabilização da epífise da cabeça do fêmur e pela salvaguarda dos vasos epifisários laterais. Durante a adolescência, o tubérculo sofre uma redução de altura e perímetro, o que pode causar instabilidade local e aumentar o risco de necrose. 6 A taxa de incidência da doença é de 1 a 7 casos por 100 mil pessoas, e o EECF afeta mais meninos, normalmente por volta dos 14 anos de idade. 7 A maturidade esquelética, os distúrbios metabólicos, a morfologia femoral e o índice de massa corporal podem influenciar o desenvolvimento da doença, que está frequentemente associada ao aumento do peso corporal. 8 9 A doença é mais frequente do lado esquerdo, e pode acometer os 2 lados em até 80% dos casos. O acometimento bilateral pode ser simultâneo ou ocorrer em momentos diferentes, geralmente nos primeiros 18 meses após o acometimento de um dos lados. 10 11 A intervenção cirúrgica é um tratamento bem estabelecido para a doença, e o monitoramento do quadril contralateral é crucial. Nos últimos anos, vários parâmetros radiográficos foram examinados para identificar sinais precoces de EECF no quadril contralateral; eles incluem o ângulo de Southwick, que indica o aumento da inclinação epifisária, 12 o ângulo de inclinação posterior, 13 o ângulo alfa 14 e a inclinação epifisária. 15 Embora alguns autores 16 17 defendam a fixação profilática com base em uma combinação de dados clínicos, evidências radiográficas e indicadores sociais, a avaliação do quadril não afetado continua sendo objeto de estudo. 18 Recentemente, um novo parâmetro de imagem objetivo, conhecido como sinal de radiotransparência peritubercular, foi proposto. 19 Acredita-se que esse sinal seja evidente nas radiografias desde as primeiras alterações que ocorrem no tubérculo epifisário e na metáfise correspondente. No entanto, a sua aplicação prática em ambientes clínicos ainda é incerta.

Este estudo tem como objetivo determinar se o sinal de radiotransparência peritubercular pode ser usado como parâmetro radiográfico confiável para o diagnóstico precoce e como preditor de doença no quadril contralateral em pacientes com EECF unilateral. Além disso, pretendemos avaliar se a ausência deste sinal pode prever a ausência de EECF. Por fim, a concordância interobservadores nas análises radiográficas também foi avaliada.

## Materiais e Métodos

Este estudo longitudinal retrospectivo foi realizado no Hospital Infantil Joana de Gusmão. A população do estudo foi composta por pacientes com diagnóstico inicial de EECF unilateral que não haviam sido submetidos à cirurgia do lado oposto e com acompanhamento ambulatorial mínimo de 18 meses no Serviço de Ortopedia entre 1995 e 2020. Pacientes que não apresentaram fechamento fisário nesse período foram monitorados até o fechamento completo da cartilagem trirradiada, e todos foram acompanhados até atingirem a maturidade esquelética. Os pacientes selecionados foram numerados sequencialmente de acordo com sua inclusão no estudo. Os dados foram coletados retrospectivamente de prontuários eletrônicos e físicos, conforme o instrumento de pesquisa descrito no Anexo 1 . As radiografias obtidas durante o acompanhamento foram classificadas cronologicamente em cada caso e revisadas por dois cirurgiões ortopédicos em início de carreira (revisores 1 e 2). Estes cirurgiões procuraram o sinal de lucência peritubercular conforme descrito em 2018 por Maranho et al. 19 ( Fig. 1 ). Apenas as radiografias realizadas nas incidências anteroposterior (AP) e de perfil (perna de rã) foram consideradas válidas. Os revisores 1 e 2 avaliaram a presença ou ausência do sinal em todas as radiografias. O sinal foi considerado presente se aparecesse em pelo menos uma radiografia, e ausente, se não fosse encontrado em nenhuma. As respostas foram consideradas válidas quando ambos os revisores concordaram quanto à presença ou ausência do sinal. Nos casos de discordância, um terceiro avaliador, um cirurgião ortopedista sênior, denominado revisor 3, foi consultado para a análise final. Todos os revisores conduziram suas análises de forma independente e cega. Anteriormente, todos os 3 revisores realizaram testes de confiabilidade intra e interobservadores em 2 rodadas, analisando 10% (15/115 casos) do tamanho total da amostra.

**Anexo 1:** Instrumento de pesquisa utilizado no estudo

Nome
Prontuário médico
Data de nascimento – dia/mês/ano
Idade em anos
Sexo – masculino/feminino
Cidade de origem
Comorbidade – descrição
Percentil na tabela peso x idade
Tempo de evolução dos sintomas – em dias
Trauma – presente ou ausente
Local de início dos sintomas – descrição
Data do primeiro atendimento – dia/mês/ano
Primeiro lado – direito/esquerdo
Primeiro grau lateral – 0/1/2/3
Cirurgia do primeiro lado – dia/mês/ano
Primeiro tipo de fixação lateral – *in situ* /redução
Material do primeiro lado – descrição
Segundo lado – direito/esquerdo
Segundo grau lateral – 0/1/2/3
Tipo de fixação do segundo lado – *in situ* /redução
Material do segundo lado – descrição
Sinal de radiotransparência – presente/ausente
Presença de sinal – sim/não
Tempo de acompanhamento – em meses
Complicação – descrição
Retirada de material – sim/não

**Fig. 1:** Sinal de lucência peritubercular 19 (2018).

Foram excluídos pacientes que já haviam sido submetidos à fixação em outro serviço ou à fixação profilática do quadril não afetado, aqueles que não apresentavam radiografias adequadas para revisão, ou que foram diagnosticados com alguma outra doença no quadril contralateral. Os casos que já se apresentaram com doença nos dois quadris desde o início (bilaterais) não foram considerados. Um fluxograma da seleção do paciente é mostrado na Fig. 2 .

**Fig. 2:** Fluxograma da seleção de pacientes. Foram selecionados 115 casos com base nos critérios de inclusão.

Foram realizadas análises descritivas das variáveis. As relações entre as variáveis de interesse foram examinadas por meio do teste de correlação do coeficiente de contingência C, com nível de significância de *p* < 0,01. A concordância interobservadores foi verificada por meio do coeficiente de concordância Kappa, com nível de significância de *p* < 0,01. Os dados foram analisados usando o programa IBM SPSS Statistics Subscription for Windows, versão Build 1.0.0.1406 (IBM Corp., Armonk, NY, Estados Unidos).

Este estudo foi realizado após aprovação do Comitê de Ética em Pesquisa da instituição (parecer n° 42937121.2.0000.5361). O estudo baseou-se na Resolução n ^o^ 500/16 do Conselho Nacional de Saúde (CNS), e obedeceu aos princípios éticos de beneficência, não maleficência, justiça e autonomia.

## Resultados

Este estudo incluiu 115 pacientes com diagnóstico de EECF unilateral à primeira consulta. Durante o acompanhamento, 30 pacientes desenvolveram a doença no quadril contralateral. A idade média foi de 11,81 (variação: 9–15) anos, e o período médio de acompanhamento foi de 32,8 (variação: 4–96) meses ( Tabela 1 ). Na análise de obesidade e sobrepeso, utilizamos como referência o índice peso para a idade, e referenciamos os gráficos de percentis dos Centers for Disease Control and Prevention. 20 Dois pacientes apresentavam múltiplas comorbidades (hipotireoidismo e doenças associadas à obesidade). Em relação à duração dos sintomas, verificamos que 33% dos casos eram agudos, 62%, crônicos, e 5%, crônicos e agudos de acordo com a classificação de Fahey e O'Brien. 21 A gravidade de cada quadril afetado foi medida de acordo com a classificação de quantificação do escorregamento da epífise em relação ao colo femoral, como descrita por Wilson et al. 22 Um dos casos com classificação de gravidade, um foi diagnosticado por cintilografia, e o outro foi diagnosticado com base nos sintomas de dor e recusa a suportar peso no membro acometido. O tempo médio desde a primeira consulta até o primeiro procedimento cirúrgico foi de 3,19 (variação: 0–30) dias. Nos pacientes que durante o seguimento evoluíram com a doença no quadril contralateral e precisaram de fixação, o intervalo entre as 2 cirurgias foi em média de 312 (variação: 26–810) dias. A fixação *in situ* foi o método de escolha em 86,89% dos casos. Os materiais utilizados para a fixação foram parafuso canulado ou fio metálico rosqueado (pino de Schanz). A escolha do implante variou ao longo dos anos, de acordo com o momento cronológico da realização do procedimento e a disponibilidade dos materiais de síntese no hospital. A avaliação do sinal de lucência peritubercular está descrita na Tabela 2 . O sinal estava presente em 31,3% de todos os casos. Dos 30 indivíduos que desenvolveram a doença, 21 apresentaram esse sinal. O paciente mais jovem que apresentou o sinal e evoluiu para a doença era um menino de 9 anos e 1 mês de idade, e o paciente mais velho um menino com exatamente 14 anos. Ao parear a presença ou ausência do sinal com a idade, mantivemos a hipótese de nulidade, uma vez que o comportamento da idade não difere significativamente entre os grupos que evoluíram ou não para a doença ( Tabela 3 ). Observamos correlação significativa entre a presença do sinal de radiotransparência peritubercular e a EECF contralateral ( *p* < 0,001). O coeficiente de contingência C apresentou valor de *p* de 1,06 × 10 ^−7^ ( *p* < 0,001). O limite superior do coeficiente de contingência C foi 0,707, e a correlação foi de 0,44. Os dados obtidos a partir dessas análises estão descritos na Tabela 4 .

**Tabela 1:** Dados demográficos e características clínicas da amostra do estudo

	n	Porcentagem válida**
**Sexo**		
- Masculino	76	66,1%
- Feminino	39	33,9%
**Comorbidade**		
- Obesidade	66	56,4%
- Sobrepeso	17	20%
- Outras	3	3,6%
**Primeiro quadril acometido**		
- Lado		
Direito	33	28,7%
Esquerdo	82	71,3%
- Grau*		
0	2	
1	68	
2	26	
3	15	
- Tipo de fixação		
Redução	14	
Fixação *in situ*	99	
**Segundo quadril acometido**		
- Lado		
Direito	23	76,7%
Esquerdo	7	23,3%
- Grau*		
0	11	
1	15	
2	1	
3	0	
- Tipo de fixação		
Redução	0	
Fixação	28	

**Porcentagem válida considera apenas os casos que continham informações no prontoário.

**Nota:** *De acordo com Wilson et al. ^22^

**Tabela 2:** Avaliação do sinal de radiotransparência peritubercular

Sinal de radiotransparência	Unilateral (n)	Bilateral (n)	Total (N)
Ausente	71	9	80
Presente	14	21	35
**Total**	**85**	**30**	**115**

**Notas:** Unilateral – não houve evolução para a doença; bilateral – houve evolução para a doença.

3.1 Pacientes com sinal: pareamento das idades dos acometidos (bilateral) e não acometidos (unilateral)



**Tabela 3:** Sinal de pareamento com a idade

	**N**	**Mínimo**	**Máximo**	**Média**	**Erro desvio**
Idade (meses)	36	9,08	14,00	11,9844	1,33053

3.2 Pacientes sem sinal: pareamento das idades dos acometidos (bilateral) e não acometidos (unilateral)



**Table:** 

	N	Mínimo	Máximo	Média	Erro desvio
Idade (meses)	79	9,00	15,42	12,4181	1,20627

3.3 Grupo com todos pacientes não acometidos (unilateral): pareamento das idades dos pacientes com e sem sinal



**Table:** 

	N	Mínimo	Máximo	Média	Erro desvio
Idade (meses)	82	9,00	15,42	12,3309	1,22065

3.4 Grupo com todos pacientes acometidos (bilateral): pareamento das idades dos pacientes com e semsinal



**Table:** 

	N	Mínimo	Máximo	Média	Erro desvio
Idade (meses)	30	9,08	14,67	12,1640	1,35121

**Tabela 4:** Análise estatística: associação entre a presença do sinal de lucência peritubercular e o desenvolvimento de escorregamento epifisário da cabeça do fêmur

Dados estatísticos	
Amostra (N)	115
Tempo de aparecimento do sinal	1 mês
Tempo antes do escorregamento	40 semanas
Radiografias anteroposteriores/perna de rã	85%/95%
Acurácia	80%
Sensibilidade	70%
Especificidade	83%
Valor preditivo positivo	60%
Valor preditivo negativo	89%

Nos casos em que o sinal estava presente e os pacientes desenvolveram a doença no quadril contralateral, o sinal foi observado em média 21 dias após o diagnóstico da doença no primeiro lado. O escorregamento normalmente ocorreu aproximadamente 301 dias depois. Neste grupo, o sinal foi observado com maior frequência na incidência em perna de rã (20/21 casos, 95%) do que na incidência AP (18/21 casos, 85%). A concordância interobservador entre os revisores 1 e 2 foi forte de acordo com o teste Kappa (k = 0,0801). Em 10 casos, as avaliações precisaram ser refeitas pelo revisor 3. Não houve correlação significativa na análise entre a presença ou ausência do sinal e as variáveis de interesse ( Tabela 5 ). É importante ressaltar que, apesar das diferenças visuais observadas entre os grupos com base na presença ou ausência do sinal, elas não apresentaram significância estatística. Oito casos foram excluídos pelos seguintes motivos: dois já haviam sido submetidos à fixação em outro serviço; dois apresentavam doença degenerativa no quadril contralateral (doença de Legg-Calvé-Perthes); e quatro foram excluídos por ausência de radiografias de controle de acompanhamento. Conseguimos identificar a presença e ausência do sinal, com ou sem desenvolvimento da doença, conforme mostra a Fig. 3 .

**Fig. 3:** Avaliação radiológica do sinal de radiotransparência peritubercular e desenvolvimento de escorregamento epifisário da cabeça do fêmur (EECF). ( **A** ) Presença do sinal de radiotransparência peritubercular e desenvolvimento da doença; ( **B** ) presença do sinal de radiotransparência peritubercular e ausência de doença; ( **C** ) ausência do sinal de radiotransparência peritubercular e desenvolvimento da doença; e ( **D** ) ausência do sinal de radiotransparência peritubercular e ausência de doença.











**Tabela 5:** Análise estatística entre a presença ou ausência do sinal e as variáveis de interesse

Variáveis de interesse comparadas	Teste estatístico	Grau	Significância	Conclusão
Sinal presente *versus* idade	1,073	1	0,376	Não rejeita H _0_
Sinal presente *versus* sexo	3,196	1	0,091	Não rejeita H _0_
Sinal presente *versus* comorbidade	4,487	2	0,106	Não rejeita H _0_
Sinal presente *versus* lateralidade	1,073	1	0,376	Não rejeita H _0_
Sinal presente *versus* grau do primeiro lado	3,785	2	0,151	Não rejeita H _0_

## Discussão

Embora as alterações anatômicas e histológicas da doença tenham sido extensivamente descritas em estudos anteriores, 2 3 o sinal de lucência peritubercular foi proposto há pouco tempo como um indicador para o diagnóstico precoce. 19 Esse método demonstrou resultados comparativamente superiores aos da ressonância magnética. 23 O tubérculo está comumente localizado no quadrante posterossuperior, e é mais posterior em crianças mais novas e superior em crianças mais velhas. 5 Sua função principal é dar apoio estrutural contra as forças de cisalhamento que atuam na fise femoral capital. 5 A manifestação radiográfica da ação dessas forças seria a lucência peritubercular. 19

Conforme descrito por Kleinman et al., 6 a detecção de anomalias na metáfise femoral capital (justafisária) pode ser difícil, e provavelmente reflete a resposta reparadora localizada aos mecanismos de estresse que enfraquecem a área. Song 24 sugeriu que os ortopedistas deveriam explorar novas metodologias e algoritmos para facilitar o diagnóstico e o tratamento precoces desta doença.

Este estudo teve como objetivo avaliar a aplicabilidade desse sinal. Em nossa avaliação, de 115 pacientes que atendiam aos critérios de inclusão, 30 desenvolveram EECF no quadril contralateral. O sinal esteve presente em 31% da amostra total (36/115). Desses 36 pacientes, 58% (21/36) desenvolveram a doença, ao passo que 42% (15/36) continuaram a apresentar exclusivamente a forma unilateral. Nos casos em que o sinal foi considerado ausente (79/115), a maioria (70/79) dos pacientes não desenvolveu EECF, e 11% (9/79) desenvolveram a doença. Ao analisarmos os 30 pacientes que evoluíram para EECF, encontramos o sinal presente em 70% destes casos (21/30). Em contrapartida, entre os 85 casos que permaneceram exclusivamente unilaterais (ou seja, não desenvolveram EECF), a ausência do sinal foi notada em 82% desses pacientes (70/85).

Nossos índices de sensibilidade e especificidade foram de 70% e 82%, respectivamente. Embora estes valores sejam relativamente inferiores aos relatados por Maranho et al., 19 ainda são consideravelmente altos. Em 80% dos casos, observamos o sinal nas primeiras radiografias do primeiro lado acometido, seja no pré-operatório ou no pós-operatório imediato. O índice Kappa entre os dois observadores principais atingiu um nível de concordância forte e superior de acordo com Cohen. 25 No entanto, para aumentar a fiabilidade dos resultados, um terceiro observador sênior fez mais análises.

Nossa análise apresentou algumas limitações quanto à acessibilidade às radiografias anteriores e à falta de informações em determinados prontuários. Apesar destas restrições, mantivemos um período mínimo de acompanhamento de 18 meses, uma vez que este intervalo é amplamente aceito na literatura para a ocorrência esperada de EECF no quadril contralateral. 1 9 10 Embora existam casos documentados de ocorrência de EECF após este período, não encontramos nenhum neste estudo. Tais casos foram monitorados até o fechamento completo da cartilagem trirradiada.

Outra limitação foi a ausência de posicionamento padronizado dos pacientes durante a radiografia. O posicionamento, que pode variar em graus de flexão, extensão, rotação e abdução, muitas vezes depende do nível de dor do paciente. Porém, a incidência em perna de rã, que consideramos a mais eficaz para o diagnóstico de EECF, foi menos comprometida e a que mais revelou a presença do sinal.

Acreditamos que a busca e verificação do sinal de lucência peritubercular seja um bom guia para o diagnóstico precoce, 26 pois ajuda a prevenir casos mais graves e pronunciadas. Mais estudos precisam ser realizados para determinar se as forças que atuam na fise da cabeça do fêmur podem ser consideradas responsáveis pelo sinal.

## Conclusão

Nossos achados sugerem uma correlação significativa entre a presença do sinal nas radiografias e o futuro desenvolvimento da doença no quadril contralateral do paciente. Embora existam exames diagnósticos mais precisos, como a ressonância magnética, a presença do sinal nas radiografias parece prever o desenvolvimento da doença. A concordância interobservador foi semelhante à de outros estudos, o que apoia a sua aplicabilidade na prática clínica. Assim, o sinal de lucência peritubercular surge como uma ferramenta complementar promissora no diagnóstico precoce do EECF, pois é útil para o planejamento terapêutico e viável para aplicação em larga escala na ausência de disponibilidade de exames complementares de alto custo. Embora não tenhamos encontrado nenhuma associação entre o sinal e as características específicas do paciente ou os traços da doença, aconselhamos seu uso criterioso, considerando o exame clínico e, se necessário, outros exames complementares de imagem, pois o EECF ainda pode ocorrer, mesmo na ausência do sinal.
